# Allopathy—Allopathists

**Published:** 1858-11

**Authors:** 


Allopathy—Allopathists.—What is there in a name? Much, very much, if with some philosophers we believe that names are things. In no case can a name, intended to indicate a quality or property of matter, or of a particular creed in science, ethics, religion or politics, be regarded with indifference. A term introduced to cover a proposition in any of these departments, must, if it falls short of the intention, mislead, and by conveying wrong ideas of the question involved, it serves to propagate and extend errors, both on the part of those who support and those who oppose its tendency and results. Sometimes a word is used which answers the purpose of showing what thing it is we are talking about, but not of telling the nature or qualities of the thing itself. Methodist, for example, is now well understood to designate a member of a sect of Christians; but the word conveys no idea either of the disciplines or of the doctrines of that sect; and in these matters it would be just as applicable to Episcopalians or to Roman Catholics, as to the followers of John Wesley. This very negative character of the word, like the name of an individual, has its recommendation in its not conveying imperfect or erroneous notions on the great cardinal points of doctrine. Sometimes a name is given to a body of men, with an avowed purpose of ridicule or reproach, and, as such, it may be mischievous to the parties thus named; but if they are successful in carrying out their plans, they themselves may adopt the obnoxious term, and by giving it vogue, deprive it of its sting. Such was the case with the *Sans-culottes* of the French revolution, who, although guilty of enormous excesses, and figuring as robbers and cut-throats in Paris, showed themselves to be brave soldiers and patriotic citizens, when sent to the frontiers to repel the invasion of France by the Prussians and Austrians. The title of "beggars," applied, in ridicule, by one of the members of the council of regency governing in the name of Philip II. of Spain, to the confederate nobles of Holland and the Low Countries, who had petitioned for a redress of grievances, was promptly adopted by them to designate their league; and a wallet, such as that worn by pilgrims and beggars, was emblazoned on their banners as the emblem of their party. All this misled nobody: it neither indicated a political creed, nor gave promise of the intentions of the party adopting the title and the emblem.

Among the "Fallacies of Confusion" laid down by logicians, stands at the head "that multitudinous body of fallacious reasonings in which the source of error is the ambiguity of terms; when something which is true, if a word be used in a particular sense, is reasoned upon as if it were true in another sense." Allopathy is one of those ambiguous terms, on the abuse of which we propose to offer some strictures. It is sufficiently distinctive, when it is used to designate a system of therapeutics, or a method of medical practice in direct contrast with and opposition to homœopathy; but it is misleading and false when it is intended to indicate the therapeutical doctrine and practice of regular physicians. We can conceive that there might be such persons as allopathists, but we have never known of men who were exclusively so as practitioners of medicine. The absurdity of homœopathy as an extreme and exclusive doctrine, which in theory takes but a partial and necessarily imperfect view of the premises, and which, in practice, carries out its consequences in a lame and contradictory manner, cannot be rectified by the adoption of allopathy as its opposite, and which would consist in another extreme and exclusive creed. Neither the dogma of the former *similia similibus*, nor that of the latter *contraria contrariis*, can furnish safe indications for the employment of the various curative means with which experience has made us acquainted.

A quaker would think it very illogical, if, because he objected to wearing black, he were to be told that he was, therefore, pledged to robe himself all in white. A hater of monarchical despotism is not, on this account, pledged in support of democracy; nor is he who abhors assassination to be classed among the supporters of tyranny.

As to the theory of homœopathy—like removed by like—the replacing of a series of symptoms indicative of disease by another series of symptoms of an analogous character induced by remedies, it is carried out by the regular practitioner in many cases: but he does so by the administration of medicines which are appreciable by weight and measure, and which produce, in their immediate operation, sensible effects; in this respect, differing essentially from the exclusive homœopathist, who gives infinitesimal doses, the minuteness of which goes beyond the power of imagination. There are cases in which we sometimes prescribe like against like, as when we remove the pain of pleurisy by inducing the pain from a blister, and stop diarrhœa by a purgative, and vomiting by an emetic; while, on the other hand, we prescribe, allopathically, for other stages of these diseases, venesection and sedatives in pleurisy, and astringents and opiates in diarrhœa. Mere allopathy, as meant to embody a system of the practice of medicine, is not less absurd than mere homœopathy. Fortunately for the sake of common sense and logic, and the health of the people, only one of these absurdities, the latter, has taken possession of many minds, as the fundamental doctrine of their medical creed. It would doubtless suit well the views of the homœopathists to see the regular members of the profession take up a directly antagonistic, though necessarily a false position as exclusively allopathists, so that the public, only seeing two theories, or rather dogmas, is left to choose at random between them; with, however, a natural leaning in favor of the one which promises a cure by the easiest and most pleasant means.

In the name of the regular profession of medicine and of medical philosophy, and bearing in mind the experience of the past and observation of the present, we protest against receiving from an inimical, a prejudiced, and an incompetent party, a title which professes, but fails by its ambiguity, to designate our medical creed, and to shape our practice under it. We can no more admit the right of homœopathists to assign us the false position of allopathists, than we can that of the vegetarians from whom we may differ, to assume that we belong exclusively to the *carnivoræ*.

We would entreat our professional brethren, everywhere, if they value sound medical philosophy and entire freedom of therapeutical selection, to resist on all occasions this insidious attempt of their enemies, and never to allow themselves to be misnamed or nicknamed allopathists.

